# 0082. Early circulating lipid and cytokine profiles prognosticate in a rat model of faecal peritonitis

**DOI:** 10.1186/2197-425X-2-S1-P2

**Published:** 2014-09-26

**Authors:** W Khaliq, M Singer

**Affiliations:** Bloomsbury Institute of Intensive Care Medicine, University College London, London, UK

## Introduction

In stress states, catecholamines induce lipolysis and insulin resistance with hyperglycaemia. Lipid profiles differ between surviving and non-surviving septic patients [1, 2] but, hitherto, little attention has been paid to this finding and its significance remains unknown. We used a previously characterized 72h fluid-resuscitated rat model of faecal peritonitis where prognostication can be made with high sensitivity and specificity as early as 6h from heart rate or stroke volume [3].

## Objectives

To determine the relationship between early changes in plasma cytokine and metabolic profiles, and their prognostic significance.

## Methods

Under general anaesthesia male Wistar rats (325±15g) underwent tunneled insertion of carotid arterial and jugular venous lines, followed by i.p. injection of 4µl/g faecal slurry. They were then woken and attached to a swivel-tether system allowing free movement in their cage with, from 2h, fluid resuscitation (1:1 mix of 5% dextrose:Hartmann's) at 10ml/kg/h. An echocardiography-measured HR cut-off of 460 bpm was used to classify animals into predicted survivors or non-survivors. At 6h, animals were sacrificed for blood and tissue sampling. We here report plasma levels of IL-6, IL-10, and a metabolic profile using blood gas analysis, ELISA and enzymatic colorometric testing.

## Results

At 6h the animals manifested only mild clinical features of illness, however significant differences were seen in IL-6 and all lipid measurements between predicted survivors and non-survivors. Glucose, lactate and IL-10 levels did not differ. Table 1

**Table 1:** Table 1

	Predicted survival (n=6)	Predicted non-survival (n=6)
**IL-6 (ng/mL)**	0.94 ± 0.23	3.70 ± 0.83*
**IL-10 (ng/mL)**	0.33 ± 0.05	0.30 ± 0.12
**Glucose (mmol/L)**	6.8 ± 0.7	6.9 ± 0.6
**Lactate (mmol/L)**	1.9 ± 0.5	1.6 ± 0.5
**HDL cholesterol (mmol/L)**	0.88 ± 0.04	0.73 ± 0.07*
**LDL/VLDL cholesterol (mmol/L)**	0.50 ± 0.03	0.39 ± 0.03*
**Triglyceride (mmol/L)**	1.12 ± 0.04	0.75 ± 0.08*

*[Data shown as median* ± SE; * p<*0.05]*

## Conclusions

In this long-term rat model of faecal peritonitis, predicted non-survivors had a significantly different IL-6 and lipid profile as early as 6 hours after sepsis. IL-6 impacts on lipid metabolism [4] but the relationship in sepsis has not, to our knowledge, been previously described. The impact of early hypolipidaemia on outcome warrants further investigation.
